# Immuno-analysis of microparticles: probing at the limits of detection

**DOI:** 10.1038/srep16314

**Published:** 2015-11-10

**Authors:** Sharissa L. Latham, Natalia Tiberti, Naveena Gokoolparsadh, Karen Holdaway, Pierre Olivier Couraud, Georges E. R. Grau, Valery Combes

**Affiliations:** 1 Vascular Immunology Unit, Sydney Medical School, The University of Sydney, NSW, Australia; 2 Institute for Biophysical Chemistry, Hannover Medical School, Hannover, Germany; 3 Australian Centre for Microscopy and Microanalysis, The University of Sydney, NSW, Australia; 4 Beckman Coulter Australia, 23-27 Chaplin Drive, Lane Cove West, NSW 2066, Australia; 5 Inserm, U1016, Institut Cochin, Paris, France; 6 Cnrs, UMR8104, Paris, France; 7 Univ Paris Descartes, Paris, France; 8 Faculty of Science, School of Life Sciences, University of Technology Sydney, NSW, Australia

## Abstract

Microparticle (MP) research is clouded by debate regarding the accuracy and validity of flow cytometry (FCM) as an analytical methodology, as it is influenced by many variables including the pre-analytical conditions, instruments physical capabilities and detection parameters. This study utilises a simplistic *in vitro* system for generating MP, and through comparative analysis with immuno-electron microscopy (Immuno-EM) assesses the strengths and limitations of probe selection and high-sensitivity FCM. Of the markers examined, MP were most specifically labelled with phosphatidylserine ligands, annexin V and lactadherin, although only ~60% MP are PS positive. Whilst these two ligands detect comparable absolute MP numbers, they interact with the same population in distinct manners; annexin V binding is enhanced on TNF induced MP. CD105 and CD54 expression were, as expected, consistent and enhanced following TNF activation respectively. Their labelling however accounted for as few as 30–40% of MP. The greatest discrepancies between FCM and I-EM were observed in the population solely labelled for the surface antigen. These findings demonstrate that despite significant improvements in resolution, high-sensitivity FCM remains limited in detecting small-size MP expressing low antigen levels. This study highlights factors to consider when selecting endothelial MP probes, as well as interpreting and representing data.

The biological significance of membrane microparticles (MP) is increasingly demonstrated across a broad range of pathologies, where their elevated levels in patient plasma are indicative of cellular stress and disease severity. MP are distinguished from other extracellular vesicles (i.e. exosomes and apoptotic bodies) by their derivation from the cells plasma membrane and their submicron size range. Endothelial MP have displayed diagnostic potential in cases of preeclampsia, Churg-Strauss syndrome and pulmonary hypertension, along with prognostic value as predictors of atherosclerotic events, cardiovascular risk, haemodynamic stress and heart failure^1^,^2^,^3^,^4^,^5^.

Flow cytometry (FCM) is the most commonly employed method for MP analysis. Notable benefits include that large MP numbers may be analysed quickly, size gating provides a way for eliminating large cellular debris and other cell types from the analysis without the need for MP purification, and that numerous antigens (Ag)/markers can be detected simultaneously on a single MP. Appropriate probe selection poses a considerable challenge for MP quantification. Conventionally, annexin V conjugates have been used as they bind phosphatidylserine (PS), a negatively charged phospholipid exposed on the plasma membrane during vesiculation^6^,^7^,^8^,^9^. More recently lactadherin has become the preferred probe for this MP hallmark as it is reported to be more sensitive to PS and is compatible with calcium chelators such as EDTA^10^,^11^,^12^,^13^. Numerous reports have however detailed that large proportions of MP are in fact PS negative and do not bind either annexin V or lactadherin, raising concerns for the use of PS labels in MP detection and analysis^13^,^14^,^15^,^16^,^17^,^18^.

An alternative labelling approach is to target surface Ag that are expressed on the cell membrane when MP are shed. This method is favourable for plasma samples where MP are derived from many cellular origins and it is essential to discern their lineage. Endothelial surface markers utilised to differentiate endothelial MP include CD31 (PECAM-1), CD51 (Integrin αV), CD54 (ICAM-1), CD62E (E-selectin), CD105 (Endoglin), CD106 (VCAM-1), CD144 (VE Cadherin), CD146 (MCAM)^14^,^19^,^20^. Of these, CD105 and CD144 are the only constitutive markers not expressed by other circulating cell types. Whilst each of these markers is routinely used for FCM, no thorough morphological characterisation and quantitative assessment has been undertaken to assess probe suitability for MP analysis.

Along with probe selection, MP analysis is also greatly influenced by other pre-analytical and analytical variables, although substantial efforts have been made to standardise methodologies such as sample collection, storage, immuno-phenotyping and cytometer calibration across the field^20^,^21^,^22^,^23^. The primary disadvantage of standard FCM detectors remains their limited ability to resolve events below 0.5 μm, differentiate background noise from true MP and distinguish coincident events. Various reports have demonstrated that technological advances in new generation cytometers and modified approaches in detection triggering improve the sensitivity of this method^17^,^24^,^25^,^26^. Robert *et al.* specifically showed that the high-sensitivity FCM achieved with the Gallios cytometer, employing a wide^2^ forward scatter detector to improve dynamic range, permitted the quantification of small-size MP. Their findings have however not be corroborated by additional methods, and as such the limitations and strengths of these technological advances remain undescribed.

The study aims to assess two specific concerns regarding MP quantification; probe selection and the limitations of MP detection in high-sensitivity FCM. To address this we 1) investigated select constitutive and inducible endothelial MP probes by FCM, assessing MP numbers and surface expression levels, 2) phenotypically characterised MP and marker expression by immuno-electron microscopy (immuno- EM) and 3) compared quantitative assessments of the two sets of findings.

## Results

### FCM characterisation of endothelial MP

**Figure 1:**
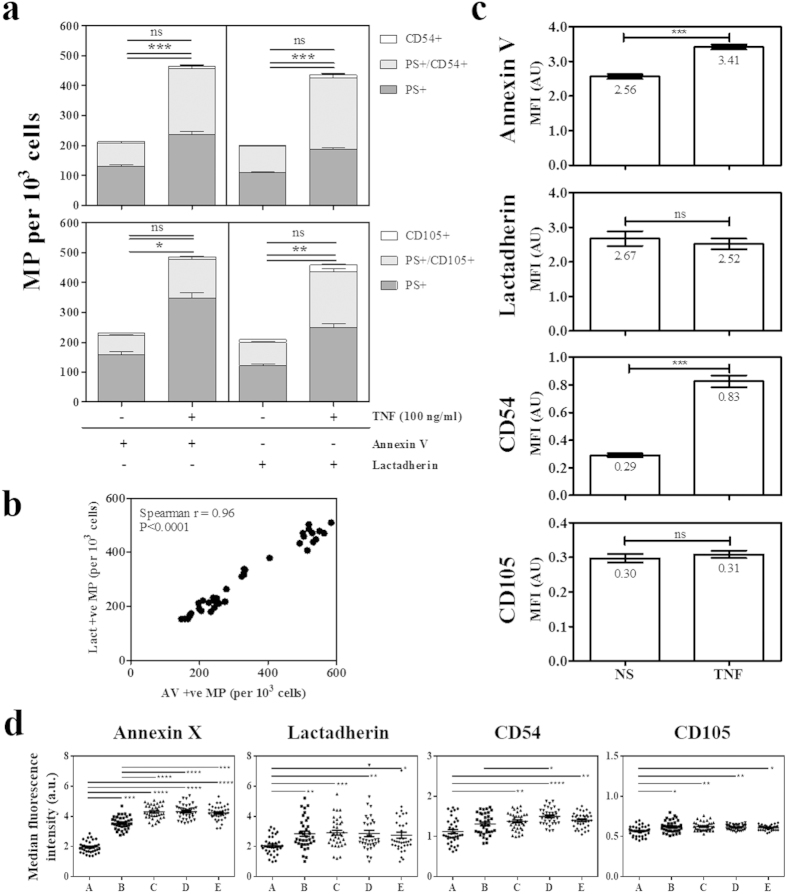
Endothelial MP quantitation and characterisation by FCM. Culture supernatants were collected from control or TNF activated (100 ng/ml overnight) hCMEC/D3 cells and labelled with either annexin V-FITC or lactaherin-FITC in combination with either CD54-PE or CD105-PE. (**a**) The total numbers of MP per 10^3^ cells are shown with dark grey bars representing the levels of PS + MP, light grey bars representing double + MP and white bars indicating the numbers of Ag + MP. Annexin V is shown on the left hand side of each graph vs lactadherin on the right, CD54 is on top and CD105 below (mean ± SE; *p < 0.05, **p < 0.01, ***p < 0.0001). (**b**) The numbers of annexin V and lactadherin positive MP per 10^3^ cells were compared between the 6 replicate wells analysed over 3 individual experiments (n = 18). Spearman’s test shows a significant correlation between the two PS labels (r = 0.96; p < 0.0001). (**c**) Mean fluorescence intensity (MFI) data for each individual label were pooled (n = 36) and differences between resting (NS) and TNF MP were assessed (mean ± SE; ***p < 0.0001). (**d**) Median fluorescence intensity for arbitrary size gates (**a**–**e**) (smallest to largest) for each individual label (mean ± SE; *p < 0.05, **p < 0.01, ***p < 0.001, ***p < 0.0001).

MP in resting and TNF-stimulated conditions were double labelled for PS (annexin V or lactadherin) and a surface Ag (CD105 or CD54). MP numbers positive for either or both markers were recorded and are represented in Table 1 along with the calculated percentages of positive MP for the various markers. TNF stimulation significantly increased the numbers of MP detected by the PS markers whether they appeared single or double stained (Fig. 1a). In all combinations, both lactadherin and annexin V detected similar numbers of MP as shown in Fig. 1a and Table 1. Moreover, a highly significant correlation between these numbers was observed (Fig. 1b).

A small minority of the CD54 + MP were labelled with CD54 only, both in resting and stimulated conditions (~4% for all combinations), while in resting conditions an average of 95% and 96% of CD54 + MP were double labelled with annexin V and lactadherin, respectively. However, MP positive for PS only represented 62% and 59% of the total MP detected with annexin V and lactadherin, respectively. These percentages, in stimulated conditions, were 51.5% and 43.7% for annexin V and lactadherin, respectively. CD105 gave a similar labelling profile with a large majority (88–92% depending on the combinations) of CD105 + MP being double labelled with either PS marker. The proportion of PS marker alone was more important within the CD105 staining (72%).

It is important to note that the percentage of the labelled population represented within the total number of events detected by the cytometer could vary significantly depending on the combinations of markers used (Table 1). CD54 and CD105 positive MP could represent as little as 5 and 3% of the total events when observed in combination with annexin V and lactadherin.

The level of fluorescence of the MP population in the resting vs stimulated conditions was then analysed regardless of whether they were double or single stained (Fig. 1c). The annexin V+ population showed a significant increase in mean fluorescence intensity (MFI) following stimulation (p < 0.0001), while the other PS marker, lactadherin showed no change. Among the surface Ag, CD54 MFI was significantly increased (p < 0.0001), while CD105 remained unchanged. When arbitrary size gates were defined and the corresponding median fluorescence recorded we observed that, in stimulated conditions, for all surface markers the level of fluorescence was significantly higher as relative size increased (Fig. 1d). The greatest differences in the level of fluorescence between relative size gates were observed with annexin V labelling with a 2.17-fold increase between gates A (smallest) and E (largest). Lactadherin had a smaller fold change between gates A and E of 1.33-fold, with greater inter-size variation seen. Although significant, levels of fluorescence of CD54 and CD105 only increase by 1.25- and 1.09-fold, respectively.

### Endothelial MP size distribution

**Figure 2:**
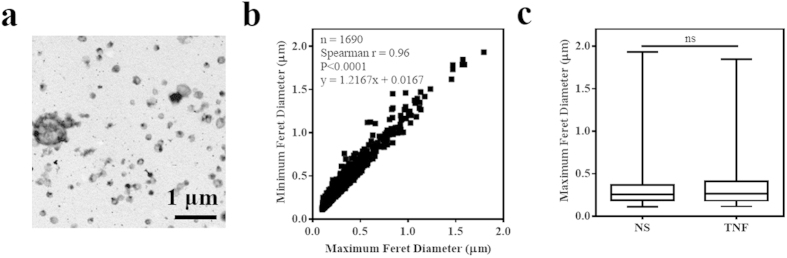
Quantitative EM shows no influence of vesiculation agonist on MP size. (**a**) Representative transmission electron micrograph of purified, negatively stained MP collected with the JEOL 1400 TEM at 20 000 X Mag. (**b**) Minimum vs maximum Feret diameters (μm) for all MP analysed (n = 1690) with Image J software, illustrating overall endothelial MP size distribution. A significant correlation was confirmed between the two parameters (Spearman’s r = 0.96; p < 0.0001). (**c**) No significant differences in the distribution (Median, 25–75% percentile and range) of maximum Feret diameters (μm) between resting (NS) and TNF MP were observed (Mann Whitney test; p = 0.38).

Purified MP from resting and TNF-activated hCMEC/D3 were subsequently examined by immuno-EM. Heterogeneity in intra-sample size and density is demonstrated in Fig. 2a, a representative low magnification micrograph of negatively stained MP from TNF-activated hCMEC/D3. Across the 16 experimental groups in this EM analysis, 6.87% ± 5.07 (SD) of events were eliminated for falling under the 0.1 μm MP size limit and 0.14% ± 0.41 exceeded the 2 μm range. Of the 1690 events which fell within the set dimensional boundaries, 2.20% ± 1.76 were within the 1–2 μm range. MP diameters ranged from 0.11–1.93 μm and 0.10–1.79 μm for the maximum and minimum Feret values, respectively. A significant correlation between these measurements was observed (Fig. 2b, Spearman’s coefficient r = 0.96, p < 0.0001), demonstrating that we are examining a distinct population of vesicles with proportional maximum and minimum sizes. Furthermore, the equation of the best fit line, where y = 1.2167x + 0.0167, supports that these MP are spherical and not augmented. The respective mean, median and mode of the maximum (0.34; 0.26; 0.19) and minimum (0.26; 0.20; 0.15) diameters (in μm) indicate that MP from the hCMEC/D3 are most concentrated in the lower end of the size range. No significant differences in MP size distribution were observed between the resting and TNF activated conditions (Fig. 2c; Mann-Whitney test, p = 0.38).

### MP characterisation by quantitative immuno-EM

**Figure 3:**
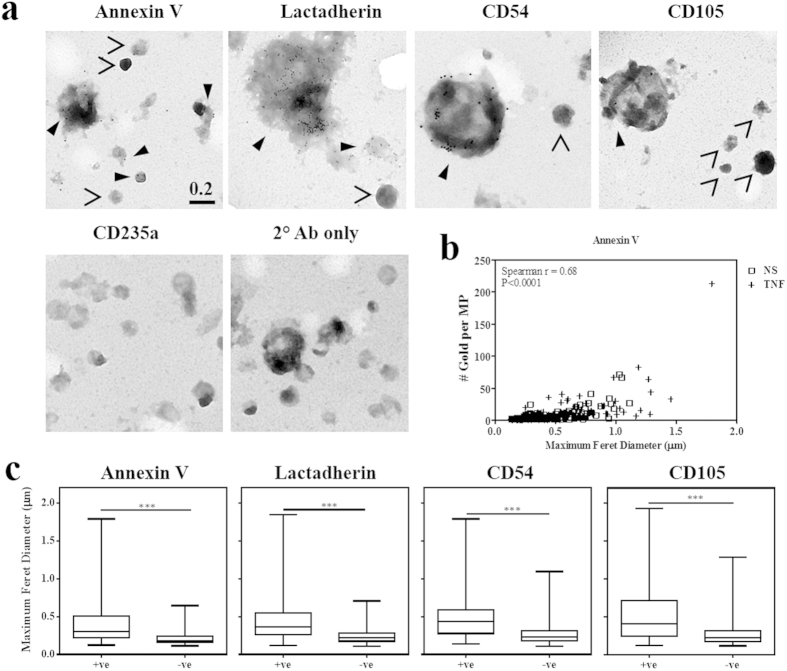
A direct correlation between MP size and marker expression. (**a**) Immuno-gold labelled MP grids were imaged at 60 000X Mag with the JEOL 1400 TEM. All images are to the same scale where scale bars are measured in micron. Closed arrow-heads indicate gold positive MP whilst open arrow-heads indicate gold-negative MP. (**b**) Spearman’s correlation shows a significant relationship between the size of MP, measured as the maximum Feret diameter (μm), and the number of gold particles bound per MP for the annexin V label (r = 0.68; p < 0.0001). (**c**) The median size, measured as the maximum Feret diameter in μm, was compared between gold-positive and gold-negative MP for each of the markers of interest (Mann Whitney test; ***p < 0.0001).

The four markers of interest, annexin V, lactadherin, CD54 and CD105, were examined separately on the MP surface by immunogold labelling. Positively labelled events are shown in Fig. 3a with closed arrow-heads, whilst MP negative for gold particles are marked with open arrow-heads. CD235a (Glycophorin A), an erythrocyte specific Ag (used as an isotype-matched control), and the secondary Ab alone showed no non-specific gold-labelling amongst samples. Quantification of gold particles showed a significant correlation between elevated numbers of gold bound per MP and increased MP diameter. This was true for each of the markers examined, where the Spearman correlation test gave r values of 0.68, 0.51, 0.48 and 0.40 for annexin V, lactadherin, CD54 and CD105 respectively, and p<0.0001 in each case (see Fig. 3b for annexin V # gold vs diameter correlation data).

Annexin V positive MP made up 64% ± 16 of the 552 events counted, with a median diameter (Feret’s maximum) of 0.30 μm (range: 0.12–1.79 μm), compared with the negative fractions which ranged from 0.11–0.65 μm (median: 0.18 μm). A similar trend is observed with lactadherin, where PS positive MP comprise 62.4% ± 5 of the total MP, ranging in maximum diameter from 0.12–1.85 μm (median 0.37 μm), compared with the negative fractions which had a median diameter of 0.17 μm (range: 0.11–0.70 μm). No differences between the percentages of PS positive MP were observed between resting and TNF-stimulated. Differential surface Ag expression between resting and TNF was only observed with CD54 labelling, where 22% ± 4 and 38.3% ± 11 of total MP were Ag positive, respectively. CD54 negative MP were 0.23 μm in diameter (range: 0.1–1.10 μm) while positive MP were 0.43 μm (range: 0.14–1.79 μm). Only 19.4% ± 3.00 of MP were CD105 positive across three experiments. Consistent with the other markers, MP positive for an Ag had a larger size distribution (median: 0.41 μm and range: 0.12–1.93 μm) compared with the Ag negative population (median: 0.23 μm and range: 0.1–1.10 μm). The Mann-Whitney test confirmed this significant size difference between gold-positive and negative MP, with p < 0.0001 for each of the markers examined (Fig. 3c).

The numbers of gold particles bound to resting (NS) *vs* TNF derived MP (Table 2) was tested with the Kolmogorov-Smirnov test for frequency distribution. No significant differences between the two distributions were observed for lactadherin (p = 0.734) or CD105 (p = 0.998). Distinct variations were however observed in the annexin V and CD54 labelled samples, where higher numbers of gold were associated with MP from TNF activated hCMEC/D3 (p = 0.010 and p < 0.0001, respectively).

### Comparative FCM and immuno-EM of dual labelled endothelial MP

**Figure 4:**
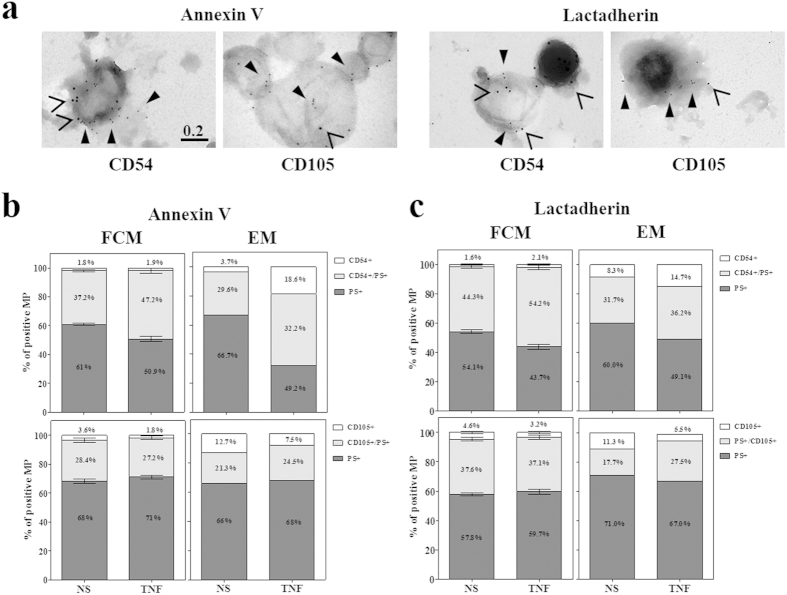
Comparative dual labelled FCM and immuno-EM show distinct differences in the percentage of Ag+ MP. (**a**) Purified MP samples dual immuno-labelled with either annexin V or lactadherin (10 nm gold, closed arrowheads) followed by anti-CD54 or CD105 Abs (15 nm gold, open arrowheads) were negatively labelled and imaged at 100 000 X Mag on the JEOL 1400 TEM. (**b,c**) The percentages of PS+ (dark grey), double + (light grey) and Ag + (white) were determined from the total numbers of MP analysed for each marker combination and for both the FCM (n = 3) and immuno-EM (n = 1) experiments. CD54 data is shown on the top row whilst CD105 is below for both annexin V (**b**) and lactadherin (**c**) labels.

PS and surface Ag expression were examined in combination with a dual immunogold labelling method utilising 10 and 15 nm gold particles. Figure 4a shows representative images of MP from TNF activated hCMEC/D3 dual tagged for annexin V or lactadherin (10 nm gold, closed arrow-heads), and CD105 or CD54 (15 nm gold, open arrow-heads). The numbers of PS +, double +, and Ag + MP were enumerated and expressed as a percentage of the total number of positive MP counted (Fig. 4b,c; right EM column). A comparison of these with the FCM data (Fig. 4b,c) show that in each sample PS labels the majority (>75%) of marker positive events. The trend for CD54+ MP to increase between resting and TNF conditions is consistent between FCM and immuno-EM, as is the percentage of CD105 which is unchanged by TNF activation.

The striking and consistent difference between the two analytic methods is the higher proportion of MP which are solely labelled for the Ag in the immuno-EM experiments. These MP are examined further in Table 3 which summarises their size distributions (range of maximum Feret diameter in μm) and the number of gold bound per MP. The median diameter for the CD54 labelled MP is 0.273 μm and median number of gold bound was 2, whilst a median of 1 gold was bound in the CD105 only population which had a median diameter of 0.25 μm.

## Discussion

The limitations of MP detection are persistently being tested by improvements to the physical components and analytical parameters of new generation flow cytometers. Improved sensitivity of the Gallios FCM in detecting MP has been shown previously^24^. Employing the wide^2^ FS detector with triggering events set on the SS channel improved the dynamic range of detection and reduced the resolution limit to ~300 nm. Whilst the authors of this work sought to demonstrate applicability of this high-sensitivity system in clinical settings, our work aimed to provide novel assessment of a selection of currently employed probes and in turn assess the strengths and weaknesses of high-sensitivity FCM.

The technically challenging and laborious nature of quantitative immuno-EM makes it a less favourable method for routine MP analysis. The study employed a whole mount negative staining method adopted from immuno-EM works used to validate Ag expression on MP^27^,^28^, and is comparable to studies specifically examining exosomes^29^,^30^. The primary benefit of this technique is that MP can be visualised in their entirety within 2-dimensions, without the need for resin embedding and sample sectioning, which is not only time consuming but can affect both image interpretation and gold quantification^31^,^32^. Drawbacks include the use of chemical fixation, which compromises membrane integrity, along with the low labelling efficiency of immuno-gold tagging. Although efficiency is estimated to be as low as 10–15% it is shown to be constant and reproducible if sample processing is standardised^33^,^34^. This means that whilst true Ag levels cannot be quantified, the relative expression between different experimental conditions and gold tags can be evaluated.

Our analysis demonstrated that of the MP probes examined, PS markers labelled almost all of the antibody fluorescent positive MP events, making PS the most efficient MP label in this study. Although other immuno-EM studies of annexin V did not quantify the numbers of gold particles bound our results are qualitatively comparable with that of Heijnen *et al.* who utilised a similar methodology. Arraud *et al.* alternatively demonstrate that MP were either heavily labelled or not labelled at all. We believe this difference in labelling density to our study is due to the 1) their annexin V being pre-conjugated to gold nanoparticles and 2) their use of cryo-EM sample preparation and imaging methods which better preserve the membrane integrity.

While high-sensitivity FCM indicates that PS positive MP account for only ~30–55% of the total MP population depending on the vesiculation stimulus, immuno-EM data determine this level to be higher at 62–65%. These relative PS-positive/-negative proportions vary extensively throughout the MP literature. For platelet MP as few as 15–20% express PS on their surface despite the majority of platelets being PS positive^13^,^15^,^16^,^17^. Arraud *et al.* estimate that approximately 50% of spherical extracellular vesicles (0.03–1 μm) were PS positive^18^. Nielsen *et al.* reported that PS positive MP accounted for 74% of the total endothelial MP levels measured, although they also suggested there may have been contamination with platelet MP due to CD31+/CD42- labelling^17^. A stark difference is that in our relatively ‘pure’ system this measure of positivity vs negativity cannot be influenced by other MP types, but rather only by the quality of the buffer (i.e. filtered vs. unfiltered PBS) and antibody (a consequence of not washing the unbound antibodies). Chandler *et al.* similarly observed that not all negative MP events appeared to have cellular origins^16^. As such it is necessary to comment one should take care when reporting percentage positivity/negativity FCM results. Reporting the absolute numbers of positive MP per volume of plasma would overcome this.

By high-sensitivity FCM, annexin V and lactadherin measured comparable levels of absolute MP numbers. Hou *et al.* report that lactadherin identifies higher MP levels than annexin V, however this observation is made after several days of storage and not on fresh samples^35^. The prominent difference that we observed between the two ligands was their labelling intensity in resting versus TNF conditions. By both FCM and immuno-EM, MFI and gold values indicated that annexin V labelling was higher on TNF MP, whilst lactadherin labelling was unchanged. The differences could not be accounted for by changes in size distribution between the two stimuli. Both ligands showed increased labelling on larger MP, as expected, however measuring the median fluorescence intensity relative to arbitrary MP size indicated that this difference is more pronounced with annexin V compared to lactadherin. In the literature it has been shown that annexin V clustering results in its preferential binding to flat membranes and that a threshold of PS must be met for adequate binding, whilst lactadherin has a preference for sharp curvatures and binds PS proportionately to the levels available^11^,^36^,^37^,^38^. Therefore, from our results we propose that the increased labelling intensity of annexin V following TNF activation may be explained by, 1) the increased availability of larger and therefore flatter MP, and 2) discrete changes in PS exposure at the membrane surface which exceed the required threshold.

In this system, CD105 labelled MP represent a modest 30% of the total marker positive MP population, whilst CD54 positive MP encompass between 40–60% depending on the activation stimuli. Thus, even in resting condition, on this particular immortalised cell line, CD54 is more highly represented on the MP than the constitutively expressed CD105. Consistency in CD105 expression between stimuli and upregulation of CD54 following TNF activation were well demonstrated by MFI measurements, observations similarly described by Jimenez *et al.*^14^. The low FCM detection of Ag-labelled MP was not unexpected, as various papers have described similar findings^39^. For CD105, the relatively low numbers of gold particles observed on MP by immuno-EM data corroborate the low expression observed by FCM. We believe this weak staining to be a true representation of CD105 expression as a number of antibody clones were tested and the same fixation and staining conditions were suitable for the other markers examined.

Comparative assessment of FCM and immuno-EM data indicates that the greatest variation between the two quantitation methods exists in these Ag single labelled populations. Where they represent between 1.5–5% of the total marker positive population by FCM, immuno-EM indicates they in fact contribute between 3–19% depending on the marker and stimulus of choice. It cannot be excluded that both the difference in antibody clone (for CD105) and conjugation (for CD54) affects the binding affinity of these labels. Closer examination of these MP alone found that they were not only on the small end of the MP size range but also expressed very low levels of Ag. As size distribution experiments however clearly demonstrate no variation between stimulus and MP size in this *in vitro* system, a finding similarly has also been observed for monocytic MP^40^, it can be reasoned that the size limitation of FCM would equally impact both stimulus conditions. Thus if a constitutive marker such as CD105 was to be used to analyse MP, an accurate relative expression between conditions could be obtained. The limitation is that precise enumeration of the true MP numbers in a sample will not be accurate; however it is clear that none of the markers available will achieve this.

It may be that overcoming these FCM limitations requires alternative high throughput analysis techniques. Nanoparticle tracking analysis (NTA) is a laser based method capable of determining particle size and suspension concentration through the investigation of vesicle Brownian motions. The use of NTA as a quantitative method for MP analysis is currently gaining momentum within the field, particularly as it is able to accurately assess events within a 50–1000 nm size range, without the limitations of a detector system or refractive index mismatch^41^,^42^. The technique is however still in its infancy regarding the use of fluorescent markers, and considerable development remains for Ab labelling to be achieved with the ease observed in FCM assays.

To summarise, constitutive and PS markers should ideally be used in combination to quantitate endothelial MP as data obtained solely with Ag markers do not appear reflective of the true number of MP events. An optimal set up would address the issue of FCM sensitivity with multi-colour antibodies or monochrome composite markers^20^. Measures of MFI are more appropriate for the assessment of inducible markers and conclusions should not easily be drawn on the degree of marker ‘negativity’ without direct visual confirmation. Annexin V and lactadherin can be used interchangeably if MP raw numbers are the primary interest of the study. However if the introduction of calcium is incompatible with the system at hand (e.g. EDTA is used as an anti-coagulant) or it is necessary to evaluate the relative levels of PS between varying MP, then the use of lactadherin would be preferential.

Ultimately, probe selection for MP FCM analysis remains at the discretion of the researcher and is dependent on the overall aims, requirements and outcomes of the study. There is great necessity for researchers within the field to form standardised quantitative approaches for MP analysis. Factors that must be addressed include how we define MP (and whether this definition solely encompasses PS positive events), how we collect and store MP, which analytic parameters are used and how we report our findings. Only then can a more holistic interpretation and understanding of MP be achieved.

## Materials and Methods

### Cell Culture

The human cerebral microvascular endothelial cell line, hCMEC/D3, were a kind gift of PO Couraud (Institut Cochin, Paris, France) cultured as previously described^43^,^44^. Briefly, hCMEC/D3 were seeded onto rat tail collagen type I (Corning Life Sciences, Cambridge, MA, USA) coated plasticware and maintained in complete EBM-2 supplemented with 5% FCS, 5 μg/ml ascorbic acid (Sigma, St Louis, MO, USA), 1.4 μmol/L hydrocortisone (Sigma), CDLC (1:100 dilution; Life technologies, Carlsbad, CA, USA), 10 mmol/L HEPES (Sigma) and 1 ng/ml β-FGF (Sigma). Cells were maintained up to passage 38. Sub-confluent monolayers were activated overnight with 100 ng/ml TNF (Peprotech EC Ltd, London, UK) in complete EBM-2 medium to increase MP release.

### Flow Cytometry (FCM)

Culture supernatants were collected and homogenized by gentle pipetting to ensure even MP distribution throughout the supernatant. Samples (100 μl of culture supernatant) were stained with either annexin V-FITC (#731725, Beckman Coulter Immunotech, Marseille, France) ± CD105-PE (#A07414, Beckman Coulter) or CD54-PE (#IM1239U, Beckman Coulter) in the presence of binding buffer (#731726, Beckman Coulter), or lactadherin-FITC (#BLAC-FITC, Haematologic Technologies Inc., Essex Jct., VT, USA) ± CD105-PE or CD54-PE in the presence of filtered PBS. These antibodies/ligands were at saturating concentrations, as determined by titration. Samples were made to a final volume of 200 μl with PBS and analysed on the Gallios^™^ flow cytometer. Six replicate wells were analysed across 3 separate experiments.

Employing the wide^2^ forward scatter detector, samples were measured over 120 seconds at a medium flow rate. Instrument detection was triggered on side scatter (SS), which was set at a threshold of 6, the lowest possible value where neither the background nor the numbers of positive events were altered. The acquisition rate did not exceed 1000 events per second. A flow rate of 18.5 μl/min was determined with Flowcount beads® (Beckman Coulter) as previously described^45^.

**Figure 5:**
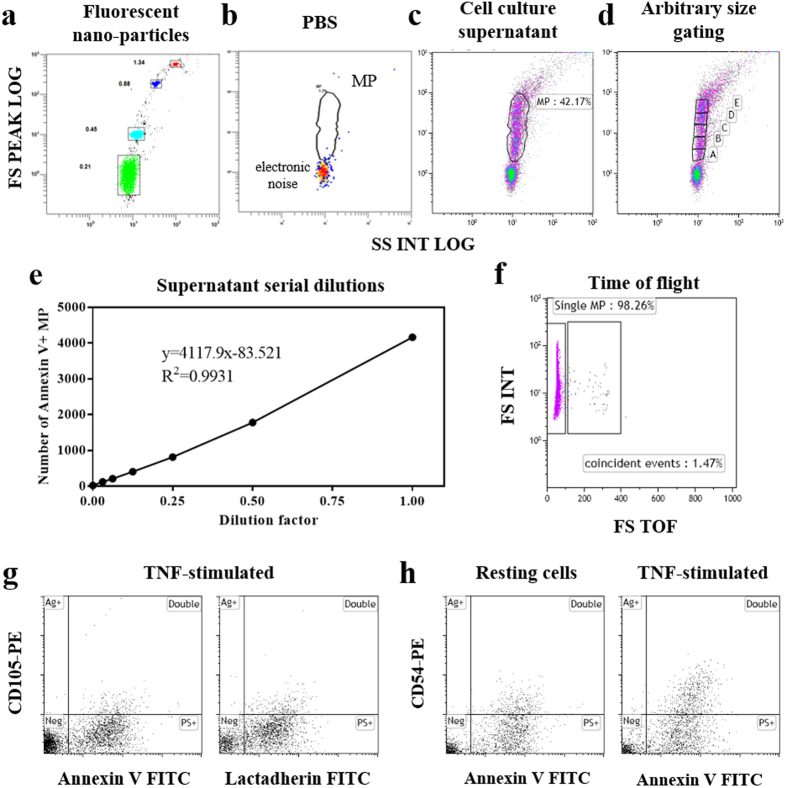
Flow cytometry setup (methods) (**a–d**) Definition of the MP gate on FSC vs SSC cytograms. (**a**) Fluorescent beads were used to set the protocol and allow evaluation of its stability. (**b**) PBS analysis demonstrates the purity of the machine where all the events detected correspond to the electronic noise of the instrument. (**c**) Analysis of a cell culture supernatant shows that the majority of the events fall with the MP gate. (**d**) Arbitrary gates (**a–e**) divide the MP gate to assess the relationship between MP size and Ag labelling. (**e,f**) examples of excluding swarm detection. (**e**) Number of MP positive for Annexin V detected in a serial dilution of the culture supernatant. (**f**) The time of Flight (TOF) parameter within a positive MP population where the left gate represent the single events and the right gate indicates the coincident events. (**g,h**) Examples of labelling combinations. CD105 vs annexin V and lactadherin on TNF-stimulated cells (**g**, left and right respectively). CD54 vs annexin V staining on resting vs TNF-stimulated cells (**h**, left and right respectively). MP were counted in the upper left quadrant for Ag only (Ag+), upper right for double labelled (double), lower right of PS marker only (PS+) and lower left for negative (Neg).

On a logarithmic scale, forward scatter (FS) was plotted against SS. Despite varying in their light scattering properties from plasma membrane vesicles, fluorescent nano-particles (Spherotech® beads, 0.21, 0.45, 0.88 and 1.34 μm in diameter) were used to set the limits of the MP size gate and ensure system stability (Fig. 5a). The electronic noise of the instrument was determined with PBS (Fig. 5b) and unlabelled cell culture supernatant, in which large debris were removed by a 1800 g 10 min spin, was used to define the analysis window of MP (Fig. 5c). In FCM, the relative size of a cell or particle is determined by the intensity of light scattering detected by the FS detector. In this manuscript we have employed both the terms “size” and “relative size” keeping this in mind. To determine the relationship between the relative size of MP and their level of fluorescence, we define arbitrary gates on the FS axis (A to E, Fig. 5d) and measured the median fluorescence intensity of each subpopulation for each marker in stimulated conditions (Fig. 1d).

Swarm detection is a major issue when small events are analysed. In addition to using a medium flow rate as described above, we have taken the following precautions to ensure that we are not counting coincident events: (1) we perform serial dilutions of our labelled supernatants or biological samples to find the optimal dilution from which we have a linear correlation between the dilution factor and the numbers of positive events detected (Fig. 5e) and (2) we use the Time of Flight (TOF) parameter of the Gallios flow cytometer, which measures the transit time of a cell or particle traversing the laser beam. This parameter allows the detection of doublets and other coincident events. Particles of a uniform size will have an equivalent TOF (Fig. 5f, single MP gate) whilst samples that are in the laser beam at the same time have less total light and take longer to traverse the laser beam (Fig. 5f, coincident events gate). Within the labelled MP, this coincident event population never exceeds 2% of the total labelled events.

Figures 5g-5h show various combinations of labelling with both PS markers and surface Ag as well as resting and stimulated conditions. FL1 (FITC) was plotted against FL2 (PE) within each sample. Samples were compensated post-acquisition against single labelled, unstained and IgG-matched controls. Buffer only controls consistently lead to 30–50 events per second. The events were disregarded in analysis as they were not positive for fluorescence and did not modulate the numbers of positive events. FL1 + /FL2- MP expressed PS alone (PS+), FL1 +/FL2+ MP were positive for both the CD marker and PS (double), whilst FL1-/FL2 + MP solely displayed the Ag on their surface (Ag+). MP numbers were expressed per 10^3^ cells. To allow reproducibility between instruments, we have analysed in our acquisition protocol Spherotech RCP-30-5A beads that contain 8 levels of fluorescence. Corresponding molecule equivalent surface fluorescence (MESF) values have been calculated for both FITC and PE channels and the corresponding data are reported in Table 4.

### Microparticle Purification

MP were purified from hCMEC/D3 culture supernatants as previously described^44^. Briefly, large cellular debris were removed at 1800 g for 10 min before supernatants were spun for 45 min at 18,000 g. MP pellets were resuspended by gently pipetting in 1 ml of PBS, sample purity was confirmed by FCM based on size and samples were furthermore spun for 45 min at 18,000 g.

### Transmission Electron Microscopy (TEM)

Purified MP were resuspended in PBS (approx. 3000 MP/μl as estimated by annexin V labelling by FCM) and adsorbed onto formvar-carbon coated nickel 300 mesh grids (5 μl/grid; Proscitech, Thuringowa, Qld, Australia) for 20 min. Labelling with annexin V-Biotin (#556417, BD Pharmingen, San Diego, CA, USA) in the presence of binding buffer or lactadherin-FITC in the presence of PBS was performed for 30 min at RT prior to fixation. Grids were fixed in 2% PFA for 15 min, washed with PBS, quenched in 0.05 M glycine for 15 min, blocked in 2% BSA in PBS for 30 min and incubated in 10 μg/ml of the following primary Ab in blocking buffer for 1 h at RT: CD105 (#sc-20072, Santa Cruz Biotechnology, Santa Cruz, CA, USA); CD54 (#IM0544, Beckman Coulter,) and CD235a (#IM2210, Beckman Coulter). Samples were washed twice in blocking buffer prior to incubation in Goat-anti-Mouse-IgG-gold 15 nm (#815–022, Aurion, Wageningen, The Netherlands) and GAM-streptavidin-gold 10 nm (#810–099, Aurion) for 1 h at RT. Grids were washed with PBS and fixed with 2% Glutaraldehyde. For lactadherin-FITC labelled samples, grids were again quenched prior to secondary conjugation with a mouse-anti-FITC-IgG-gold 15 nm (#810.233, Aurion). Samples were washed with water prior to negative staining with 2% Uranyl Acetate for 3 min at RT.

All TEM images were collected with the JEOL 1400 (JEOL, Tokyo, Japan) at 120 kV at the AMMRF facility at the University of Sydney. Single labelled samples were imaged at 60,000 X Mag whilst double labelled samples were imaged at 100,000 X Mag, as determined by optimisation experiments.

### Image Analysis

Image J software was utilised for all immuno-EM MP quantification and analysis. Briefly, MP regions of interest (ROI) were manually selected and the diameter and number of gold particles bound per MP were measured. Feret’s diameter was selected as the preferred measurement over alternatives such as perimeter and area, as MP diameter is the most common gauge of MP size in the literature. Particles with minimum or maximum Feret’s diameter values outside of the 100–2000 nm range were excluded from the analysis, regardless of gold binding.

### Statistical Analyses

Statistical analyses were performed with Graph Pad Prism 5 (Graphpad Software Inc, CA, USA) and IBM SPSS v20.0 (IBM, NY, USA) software. In each case, data normality was determined prior to application of the appropriate statistical test. MP numbers as determined by FCM were assessed with a one-way ANOVA and Bonferroni’s correction for multiple comparisons when data assumed a normal distribution or with the non-parametric Kruskal-Wallis test followed by Dunn’s test for multiple comparisons. All correlations were performed with the nonparametric Spearman’s test. All comparisons between two conditions were achieved with either the Students t test or Mann Whitney nonparametric test. Gold distributions were compared with the Kolmogorov-Smirnov test. All tests were two-tailed and the level of significance was set at p value < 0.05. All graphical representations were produced in Graph Pad Prisms 5. In the text standard error of the mean is denoted with SE and standard deviation with SD.

## Additional Information

**How to cite this article**: Latham, S. L. *et al.* Immuno-analysis of microparticles: probing at the limits of detection. *Sci. Rep.* **5**, 16314; doi: 10.1038/srep16314 (2015).

## Figures and Tables

**Table 1:** The numbers and percentages of MP as assessed by FCM.

	NS	TNF	NS	TNF
	Annexin V		Lactadherin	
CD54 and PS dual labelled samples (mean MP number/10^3^ cells ± SEM)				
CD54	3.7 ± 0.3	9.1 ± 0.8	3.2 ± 0.3	9.7 ± 1.5
PS	129.8 ± 5.8	235.1 ± 2.6*	108 ± 5	186 ± 6.9*
Double	78.9 ± 3.4	220.9 ± 14.4*	88.2 ± 3.3	239.2 ± 14.9*
Total	212.5 ± 8.6	465.1 ± 21.2*	199.4 ± 7.4	435.4 ± 16.2*
CD105 and PS dual labelled samples (mean MP number/10^3^ cells ± SEM)				
CD105	8.2 ± 0.5	8.3 ± 0.3	9.87 ± 1.8	22.5 ± 3.7*
PS	158 ± 10.8	347.3 ± 9.8*	121.2 ± 5.8	250.3 ± 12.4*
Double	61.5 ± 2.5	130.1 ± 7.4*	78.1 ± 3.6	185.1 ± 9.7*
Total	231 ± 11.3	485.8 ± 3.7*	209.2 ± 8.9	457.9 ± 23.2*
Fluorescence positivity for individual labels (% positive (% range))				
PS	31 (24–44)	48 (41–62)	44 (8–69)	52 (21–72)
CD54#	NS: 17 (5–34)		TNF: 25 (17–45)	
CD105#	NS: 13 (3–32)		TNF: 17 (9–33)	
Fluorescence positivity for multiple labels (% positive ± SEM)				
CD54/PS	37 ± 0.8	47 ± 1.6	44 ± 0.9	54 ± 1.7
CD105/PS	28.4 ± 1.5	27.1 ± 1.1	37.6 ± 1.2	37.1 ± 1.3

^*^Significant difference between non-stimulated (NS) and TNF activated conditions, Mann-Whitney test, p < 0.05.

^#^Data collated from both Annexin V and Lactadherin labelled experiments.

**Table 2:** Effect of vesiculation agonist and probe on marker expression and detection.

Labelling	NS		TNF		P Value*
	n	Median # Gold (Range)	n	Median # Gold (Range)	
Annexin V	175	2 (1–72)	184	4 (1–213)	0.01
Lactadherin	177	3 (1–172)	239	3 (1–53)	0.734
CD54	63	1 (1–12)	140	2 (1–103)	<0.0001
CD105	52	1 (1–26)	67	1 (1–19)	0.998

NS: non-stimulated; TNF: tumour necrosis factor (100 ng/ml).

n = the number of MP enumerated per condition.

Median # gold (range) = the median number and range of gold particles bound per MP.

*Kolmogorov-Smirnov comparison of gold distributions.

**Table 3:** Small size distribution and low immunogold binding in the PS-/Ag+ population.

Labelling	NS			TNF		
	n	Maximum Feret (μm)	#Gold Bound	n	Maximum Feret (μm)	#Gold Bound
AV + CD54	2	0.2–0.212	1	11	0.115–0.506	1–4
AV + CD105	6	0.19–0.415	1–4	4	0.141–0.296	1–2
Lact + CD54	5	0.204–0.438	1–2	17	0.138–0.516	1–8
Lact + CD105	7	0.184–0.266	1–6	5	0.146–0.446	1

NS: non-stimulated; TNF: tumour necrosis factor (100 ng/ml).

n = the number of double positive MP for each labelling combination.

^#^Gold Bound = the range of gold particles counted on MP in each labelling combination.

**Table 4:** Mean Equivalent Soluble Fluorochrome (MESF) values for FITC and PE in this high-sensitivity FCM system.

FITC		PE	
Relative Channel	MESF	Relative Channel	MESF
0.49	954	0.32	621
1.12	2289	0.83	1734
2.29	6351	2.27	5044
7.74	16746	6.23	14029
21.87	49198	18.05	41359
64.33	146994	56.01	130228
127.96	281594	126.77	286384

## References

[b1] Gonzalez-Quintero V. H. *et al.* Elevated plasma endothelial microparticles: preeclampsia versus gestational hypertension. Am. J. Obstet. Gynecol. 191, 1418–1424 (2004). 15507976 10.1016/j.ajog.2004.06.044

[b2] Kumpers P. *et al.* Endothelial microparticles as a diagnostic aid in Churg-Strauss vasculitis-induced cardiomyopathy. Clin. Exp. Rheumatol. 26, S86–89 (2008). 18799061

[b3] Amabile N. *et al.* Circulating endothelial microparticle levels predict hemodynamic severity of pulmonary hypertension. Am. J. Respir. Crit. Care Med. 177, 1268–1275 (2008). 18310479 10.1164/rccm.200710-1458OC

[b4] Nozaki T. *et al.* Prognostic value of endothelial microparticles in patients with heart failure. Eur. J. Heart Fail. 12, 1223–1228 (2010). 20817695 10.1093/eurjhf/hfq145

[b5] Baron M., Boulanger C. M., Staels B. & Tailleux A. Cell-derived microparticles in atherosclerosis: biomarkers and targets for pharmacological modulation? J. Cell. Mol. Med. 16, 1365–1376 (2012). 22050954 10.1111/j.1582-4934.2011.01486.x PMC3823207

[b6] Thiagarajan P. & Tait J. F. Binding of annexin V/placental anticoagulant protein I to platelets. Evidence for phosphatidylserine exposure in the procoagulant response of activated platelets. J. Biol. Chem. 265, 17420–17423 (1990). 2145274

[b7] Thiagarajan P. & Tait J. F. Collagen-induced exposure of anionic phospholipid in platelets and platelet-derived microparticles. J. Biol. Chem. 266, 24302–24307 (1991). 1662206

[b8] Dacharyprigent J., Freyssinet J. M., Pasquet J. M., Carron J. C. & Nurden A. T. Annexin-V as a probe of aminophospholipid exposure and platelet membrane vesiculation - a flow-cytometry study showing a role for free sulfhydryl-groups. Blood 81, 2554–2565 (1993). 8490169

[b9] Koopman G. *et al.* Annexin V for flow cytometric detection of phosphatidylserine expression on B cells undergoing apoptosis. Blood 84, 1415–1420 (1994). 8068938

[b10] Dasgupta S. K., Guchhait P. & Thiagarajan P. Lactadherin binding and phosphatidylserine expression on cell surface-comparison with annexin A5. Transl. Res. 148, 19–25 (2006). 16887494 10.1016/j.lab.2006.03.006

[b11] Shi J. *et al.* Lactadherin detects early phosphatidylserine exposure on immortalized leukemia cells undergoing programmed cell death. Cytometry A 69, 1193–1201 (2006). 17123296 10.1002/cyto.a.20345

[b12] Fu Y. Y. *et al.* Daunorubicin induces procoagulant activity of cultured endothelial cells through phosphatidylserine exposure and microparticles release. Thromb. Haemost. 104, 1235–1241 (2010). 20886178 10.1160/TH10-02-0102

[b13] Perez-Pujol S., Marker P. H. & Key N. S. Platelet microparticles are heterogeneous and highly dependent on the activation mechanism: studies using a new digital flow cytometer. Cytometry A 71, 38–45 (2007). 17216623 10.1002/cyto.a.20354

[b14] Jimenez J. J. *et al.* Endothelial cells release phenotypically and quantitatively distinct microparticles in activation and apoptosis. Thromb. Res. 109, 175–180 (2003). 12757771 10.1016/s0049-3848(03)00064-1

[b15] Connor D. E., Exner T., Ma D. D. & Joseph J. E. The majority of circulating platelet-derived microparticles fail to bind annexin V, lack phospholipid-dependent procoagulant activity and demonstrate greater expression of glycoprotein Ib. Thromb. Haemost. 103, 1044–1052 (2010). 20390225 10.1160/TH09-09-0644

[b16] Chandler W. L., Yeung W. & Tait J. F. A new microparticle size calibration standard for use in measuring smaller microparticles using a new flow cytometer. J. Thromb. Haemost. 9, 1216–1224 (2011). 21481178 10.1111/j.1538-7836.2011.04283.x

[b17] Nielsen M. H., Beck-Nielsen H., Andersen M. N. & Handberg A. A flow cytometric method for characterization of circulating cell-derived microparticles in plasma. Journal of extracellular vesicles 3, 10.3402/jev.v3.20795 (2014). PMC3916676 24511371

[b18] Arraud N. *et al.* Extracellular vesicles from blood plasma: determination of their morphology, size, phenotype and concentration. J. Thromb. Haemost. 12, 614–627 (2014). 24618123 10.1111/jth.12554

[b19] Jy W. *et al.* Measuring circulating cell-derived microparticles. J. Thromb. Haemost. 2, 1842–1851 (2004). 15456497 10.1111/j.1538-7836.2004.00936.x

[b20] Lacroix R., Robert S., Poncelet P. & Dignat-George F. Overcoming limitations of microparticle measurement by flow cytometry. Semin. Thromb. Hemost. 36, 807–818 (2010). 21049381 10.1055/s-0030-1267034

[b21] Gelderman M. P. & Simak J. Flow cytometric analysis of cell membrane microparticles. Methods Mol. Biol. 484, 79–93 (2008). 18592174 10.1007/978-1-59745-398-1_6

[b22] Robert S. *et al.* Standardization of platelet-derived microparticle counting using calibrated beads and a Cytomics FC500 routine flow cytometer: a first step towards multicenter studies? J. Thromb. Haemost. 7, 190–197 (2009). 18983485 10.1111/j.1538-7836.2008.03200.x

[b23] van Ierssel S. H. *et al.* Flow cytometric detection of endothelial microparticles (EMP): effects of centrifugation and storage alter with the phenotype studied. Thromb. Res. 125, 332–339 (2010). 20117824 10.1016/j.thromres.2009.12.019

[b24] Robert S. *et al.* High-sensitivity flow cytometry provides access to standardized measurement of small-size microparticles–brief report. Arterioscler. Thromb. Vasc. Biol. 32, 1054–1058 (2012). 22328775 10.1161/ATVBAHA.111.244616

[b25] Headland S. E., Jones H. R., D’Sa A. S., Perretti M. & Norling L. V. Cutting-edge analysis of extracellular microparticles using ImageStream(X) imaging flow cytometry. Sci Rep 4, 5237 (2014). 24913598 10.1038/srep05237 PMC4050385

[b26] Arraud N., Gounou C., Linares R. & Brisson A. R. A Simple Flow Cytometry Method Improves the Detection of Phosphatidylserine-Exposing Extracellular Vesicles. J. Thromb. Haemost., 10.1111/jth.12767 (2014). PMC4359678 25348269

[b27] Heijnen H. F., Schiel A. E., Fijnheer R., Geuze H. J. & Sixma J. J. Activated platelets release two types of membrane vesicles: microvesicles by surface shedding and exosomes derived from exocytosis of multivesicular bodies and alpha-granules. Blood 94, 3791–3799 (1999). 10572093

[b28] Verderio C. *et al.* Myeloid microvesicles are a marker and therapeutic target for neuroinflammation. Ann. Neurol. 72, 610–624 (2012). 23109155 10.1002/ana.23627

[b29] Van Deun J. *et al.* The impact of disparate isolation methods for extracellular vesicles on downstream RNA profiling. Journal of extracellular vesicles 3, 10.3402/jev.v3.24858 (2014). PMC4169610 25317274

[b30] Aalberts M. *et al.* Identification of distinct populations of prostasomes that differentially express prostate stem cell antigen, annexin A1, and GLIPR2 in humans. Biol. Reprod. 86, 82 (2012). 22133690 10.1095/biolreprod.111.095760

[b31] D’Amico F. & Skarmoutsou E. Quantifying immunogold labelling in transmission electron microscopy. J. Microsc. 230, 9–15 (2008). 18387034 10.1111/j.1365-2818.2008.01949.x

[b32] Mayhew T. M., Muhlfeld C., Vanhecke D. & Ochs M. A review of recent methods for efficiently quantifying immunogold and other nanoparticles using TEM sections through cells, tissues and organs. Ann Anat 191, 153–170 (2009). 19135344 10.1016/j.aanat.2008.11.001

[b33] Griffiths G. & Hoppeler H. Quantitation in immunocytochemistry: correlation of immunogold labeling to absolute number of membrane antigens. J. Histochem. Cytochem. 34, 1389–1398 (1986). 3534077 10.1177/34.11.3534077

[b34] Howell K. E., Reuter-Carlson U., Devaney E., Luzio J. P. & Fuller S. D. One antigen, one gold? A quantitative analysis of immunogold labeling of plasma membrane 5’-nucleotidase in frozen thin sections. Eur. J. Cell Biol. 44, 318–327 (1987). 2826169

[b35] Hou J. *et al.* Lactadherin functions as a probe for phosphatidylserine exposure and as an anticoagulant in the study of stored platelets. Vox Sang. 100, 187–195 (2011). 20738837 10.1111/j.1423-0410.2010.01375.x

[b36] Andree H. A. *et al.* Clustering of lipid-bound annexin V may explain its anticoagulant effect. J. Biol. Chem. 267, 17907–17912 (1992). 1387643

[b37] Shi J., Heegaard C. W., Rasmussen J. T. & Gilbert G. E. Lactadherin binds selectively to membranes containing phosphatidyl-L-serine and increased curvature. Biochim. Biophys. Acta 1667, 82–90 (2004). 15533308 10.1016/j.bbamem.2004.09.006

[b38] Hu T., Shi J., Jiao X., Zhou J. & Yin X. Measurement of annexin V uptake and lactadherin labeling for the quantification of apoptosis in adherent Tca8113 and ACC-2 cells. Braz. J. Med. Biol. Res. 41, 750–757 (2008). 18820763 10.1590/s0100-879x2008000900002

[b39] Simak J. *et al.* Elevated circulating endothelial membrane microparticles in paroxysmal nocturnal haemoglobinuria. Br. J. Haematol. 125, 804–813 (2004). 15180871 10.1111/j.1365-2141.2004.04974.x

[b40] Bernimoulin M. *et al.* Differential stimulation of monocytic cells results in distinct populations of microparticles. J. Thromb. Haemost. 7, 1019–1028 (2009). 19548909 10.1111/j.1538-7836.2009.03434.x PMC3242443

[b41] Dragovic R. A. *et al.* Sizing and phenotyping of cellular vesicles using Nanoparticle Tracking Analysis. Nanomed. 7, 780–788 (2011). 10.1016/j.nano.2011.04.003 PMC3280380 21601655

[b42] Soo C. Y. *et al.* Nanoparticle tracking analysis monitors microvesicle and exosome secretion from immune cells. Immunology 136, 192–197 (2012). 22348503 10.1111/j.1365-2567.2012.03569.x PMC3403268

[b43] Weksler B. B. *et al.* Blood-brain barrier-specific properties of a human adult brain endothelial cell line. FASEB J. 19, 1872–1874 (2005). 16141364 10.1096/fj.04-3458fje

[b44] Latham S. L. *et al.* Cooperation between beta- and gamma-cytoplasmic actins in the mechanical regulation of endothelial microparticle formation. FASEB J. 27, 672–683 (2013). 23159932 10.1096/fj.12-216531

[b45] Mfonkeu J. B. P. *et al.* Elevated Cell-Specific Microparticles Are a Biological Marker for Cerebral Dysfunctions in Human Severe Malaria. PLoS ONE 5, e13415 10.1371/journal.pone.0013415 (2010). PMC2954805 20976232

